# Dopamine attenuates ethanol-induced neuronal apoptosis by stimulating electrical activity in the developing rat retina

**DOI:** 10.1515/med-2025-1205

**Published:** 2025-06-05

**Authors:** Junde Han, Shuli Ge, Kan Zhang, Jijian Zheng, Jing Dong

**Affiliations:** Department of Anesthesiology, Shanghai Sixth People’s Hospital Affiliated to Shanghai Jiao Tong University School of Medicine, Shanghai, 200233, China; Department of Anesthesiology, Shanghai Children’s Medical Center Affiliated to Shanghai Jiao Tong University School of Medicine, National Children’s Medical Center, Shanghai, 200127, China; Department of Anesthesiology, Shanghai Cancer Center, Fudan University, Shanghai, 200032, P. R. China; Department of Oncology, Shanghai Medical College, Fudan University, No.270 Dong’An Road, Xuhui District, Shanghai, 200032, China

**Keywords:** developing neuron apoptosis, synchronized spontaneous neural network electrical activity, ethanol, dopamine, retina

## Abstract

**Background:**

Prenatal alcohol exposure causes fetal alcohol spectrum disorders (FASD), primarily through alcohol-induced apoptosis. This study explores the link between ethanol-induced neuronal apoptosis and neural network electrical activity in developing rat retinal ganglion cells, and examines dopamine’s protective effects and influence on this activity.

**Methods:**

The study employed a combination of immunohistochemical techniques, terminal deoxynucleotidyl transferase dUTP nick-end labeling assay, and electrophysiological recordings to assess neuronal apoptosis and neural network activity in the developing rat retinal ganglion cell layer. Ethanol exposure was administered to model prenatal alcohol exposure, and dopamine was applied to evaluate its protective effects.

**Results:**

Ethanol exposure was found to disrupt the spatiotemporal properties of synchronized spontaneous neural network electrical activity and partially induce neuronal apoptosis. Conversely, dopamine treatment increased the frequency of neural network electrical activity and attenuated ethanol-induced apoptosis.

**Conclusion:**

The findings suggest that ethanol disrupts neural network activity and induces apoptosis in the developing nervous system, while dopamine exerts a protective effect by modulating neural network activity and reducing apoptosis. These results contribute to understanding the mechanisms underlying FASD and offer potential therapeutic avenues for prevention and treatment.

## 1 Introduction

Alcohol exposure during pregnancy may lead to fetal alcohol syndrome (FAS), a severe form of FAS disorder, which includes a series of symptoms, such as craniofacial malformation, structural abnormalities of the nervous system, and long-term neurobehavioral disorders [1,2]. Up to 4.3–6.6% of young children have been affected by FAS in South Africa, which is similar to that in the United States and Western Europe [3–5]. The primary clinical manifestation of FAS is neurobehavioral disorders, and there is no definite conclusion about the precise mechanisms of FAS. Meanwhile, except for avoiding alcohol consumption, there are no effective clinical treatments for FAS.

Previous studies have reported that alcohol exposure during early pregnancy in both animals and humans causes irreversible damage to the central nervous system (CNS), such as the loss of neuronal mass and reduced brain volume [2,6,7]. Apoptosis of neurons and glia has been reported to occur in rodents or non-human primates due to single alcohol exposure during a period equivalent to the third trimester in humans [6,8]. Once alcohol-induced neuronal and glial apoptosis begins, a series of physiological and pathophysiological activities occur to compensate for the alcohol-induced injury and may result in permanent CNS damage. Thus, apoptosis is considered the primary mechanism resulting in FAS, and reducing alcohol-induced apoptosis may result in the prevention and treatment of FAS [9].

Ethanol-induced neuronal apoptosis in the developing neuronal system (brain, spinal cord, and retina) has been demonstrated to be associated with blocking *N*-methyl-d-aspartate (NMDA) receptors and activating gamma-aminobutyric acid (GABA) receptors [6,10]. Moreover, ethanol could induce an imbalance in the B-cell lymphoma 2 family, increasing mitochondrial membrane permeability and activating the endogenous apoptosis pathway [11]. In addition, the increased levels of reactive oxygen species, downregulation of the cyclic adenosine 3,5-monophosphate/protein kinase A (cAMP/PKA) pathway, and blockage of the insulin pathway are also associated with ethanol-induced neuronal apoptosis in developing neuronal systems [12–14]. However, the specific mechanism of ethanol-induced neuronal apoptosis remains unclear. In the developing nervous system, especially at the peak of synaptogenesis, accompanied by the maturation of the nervous system, cell signal transduction pathway, gene expression, and other physiological activity changes [15–17], the sequential switch of GABA, NMDA, and acetylcholine (Ach) receptors or subtype receptors regulates the spontaneous and rhythmic neural network activities to ensure the establishment of accurate neuronal connections and networks [18–20]. During the early development of the CNS, receptors, the synchronized spontaneous neural network electrical activity, and genes play an important role in apoptosis [21,22]. Previous studies have demonstrated that the blockade of the NMDA receptor, Ach receptor, or the synchronized spontaneous neural network electrical activity could induce neuronal apoptosis in the developing nervous system [22–25]. In addition, Feller et al. found that the application of the GABAa receptor agonist muscimol blocked the synchronized spontaneous neural network electrical activity in developing rat retina [26]. However, whether ethanol, which is an NMDA receptor antagonist, and GABA receptor agonists induce neuronal apoptosis by blocking spontaneous electrical activity in the developing nervous system remains unknown. Moreover, whether increasing the electric activity could attenuate ethanol-induced neuronal apoptosis is unclear.

In this study, the primary aim of utilizing dopamine is to investigate its protective role and underlying mechanisms in ethanol-induced apoptosis of neural cells in the developing rat retina. Dopamine, being a crucial neurotransmitter and neuromodulator, plays multiple roles in nervous system development, particularly in regulating neuronal survival, synaptic plasticity, and neural network activity [27]. Previous studies have indicated that dopamine, through activation of its receptors such as D1 and D2, can modulate intracellular signaling pathways including the cAMP/PKA pathway and calcium signaling, which are closely associated with neuronal survival and apoptosis [28]. Ethanol induces neuronal apoptosis via various mechanisms, including blocking NMDA receptors and activating GABA receptors, especially during critical developmental periods such as synaptogenesis and maturation of neural network activity. Disruptions in these receptors and spontaneous electrical activity may lead to neuronal apoptosis and abnormal network connections [29]. However, it remains unclear whether ethanol induces apoptosis by inhibiting spontaneous electrical activity and whether enhancing electrical activity can alleviate ethanol-induced apoptosis. Dopamine may exert its effects through several pathways: first, by activating its receptors to regulate neuronal electrical activity, thereby maintaining normal neural network function; second, by regulating the cAMP/PKA signaling pathway, antioxidant stress responses, or influencing mitochondrial function to mitigate the cellular homeostatic imbalances caused by ethanol [30]. Therefore, in this study, we aim to explore whether dopamine can alleviate ethanol-induced neuronal apoptosis by modulating spontaneous electrical activity or intracellular signaling pathways, thereby revealing its potential protective mechanisms in developmental neurological damage and providing new insights for the prevention and treatment of ethanol-related neurological developmental disorders.

## 2 Materials and methods

### 2.1 Animals

A total number of 69 postnatal Day 7 (P7) Sprague–Dawley rat pups were obtained from the Experimental Animal Center of Shanghai Sixth Hospital, Shanghai, China. Male and female rats were used in this study. All experimental procedures were reviewed and approved by the Animal Care Committee at the Shanghai Sixth Hospital, Shanghai Jiao Tong University School and complied with the guidelines of the Care and Use of Laboratory Animals published by the US National Institutes of Health and Association for Research in Vision and Ophthalmology Statement for the Use of Animals in Ophthalmic and Vision Research. Every effort was made to minimize the number and discomfort of the animals during all experimental procedures.

### 2.2 Tissue preparation

Whole-mount rat retinas were obtained as previously described, with slight modifications [25]. Briefly, all eyeballs were dissected swiftly using fine scissors from experimental rat pups after being sacrificed by decapitation and transferred to an ice-cold (0‒4°C) bath of an artificial cerebrospinal fluid (ACSF) (composition in mM: sodium chloride 124, potassium chloride [KCl] 3.3, potassium dihydrogen phosphate 1.2, calcium dichloride with water 2.5, magnesium sulfate 2.4, sodium bicarbonate 26, and glucose 10). A 95% oxygen (O_2_)/5% carbon dioxide (CO_2_) gas mixture was continuously bubbled into the bath. Subsequently, an incision at the edge of the cornea and sclera (approximately one-fifth of the circumference) was manipulated using ophthalmology scissors to facilitate the exposure of the ACSF and drugs into the retina. Thereafter, the eyeballs were incubated in the ACSF (37°C) equilibrated with 95% O_2_/5% CO_2_ gas mixture until the experiment was completed. All procedures were performed within 6 h.

### 2.3 Drugs and reagents

All drugs were purchased from Sigma-Aldrich (St Louis, MO, USA), unless otherwise mentioned, and dissolved in ACSF. The following drugs were used: ethanol (Aladdin, Shanghai, China), dopamine, muscimol (GABAA receptor agonist), SR95531 (gabazine, GABA_A/C_ receptor competitive antagonist), and picrotoxin (PTX) (GABAA receptor noncompetitive antagonist).

### 2.4 Electrophysiological experiments

Whole-mount rat retinas for patch-clamp recordings were obtained from P7 rat pups, as previously reported [31]. ON-alpha ganglion cells are often preferred for patch-clamp recordings at this stage due to their relatively earlier functional maturation and distinct physiological properties, which make them more suitable for studying early development in the retina. A piece of whole-mount retina was dissected from an eyeball; subsequently, the Müller cell end feet and connective tissues on the top of the ganglion cell layer (GCL) were removed with the ice-cold oxygenated ACSF. After a 30 min recovery at room temperature, the whole-mount retina was delivered and fixed to the backside of a heated bathing chamber with the sclera facet down by a small platinum ring with a nylon mesh. In the chamber, the retina was perfused constantly with the oxygenated ACSF (2–4 mL/min) at 32–35°C. Electrophysiological whole-cell patch-clamp recordings were obtained from GCL cells using Axopatch 700B amplifiers and pCLAMP 9 software (Axon Instruments, Inc., Union City, CA, USA) under an upright microscope equipped with a 40× water-immersion objective lens (BX50WI, Olympus USA, NY, USA). The borosilicate glass pipettes (B15023F, Wuhan microprobe Scientific Instrument, China) were pulled (P-97; Sutter, USA) with a resistance of 3–5 MΩ and filled with a pipette solution containing (in mM) the following: KCl 150, magnesium chloride 2, ethylene glycol tetraacetic acid 2, 4-(2-hydroxyethyl)-1-piperazineethanesulfonic acid 10, and disodium adenosine 5′ triphosphate 2 at pH = 7.2, adjusted with a Tris base. After obtaining successful seals of 1 GΩ in 30 s or less, the current responses to pharmacological manipulations were recorded at a holding potential of –60 mV under the voltage clamp, filtered at 1 kHz, and sampled at 5 kHz.

### 2.5 Cleaved caspase-3 immunohistochemistry

After incubation with or without ethanol and/or GABAa agonist/antagonist in the ACSF (37°C) equilibrated with 95% O_2_/5% CO_2_ gas mixture for 5 h, the eyeballs were fixed in 4% paraformaldehyde for 1 h. Retinas were then dissected and fixed overnight. Following dehydration with an ethanol gradient, the fixed retinas were paraffin infiltrated and embedded in paraffin blocks for long-term preservation. Retinas were cut into 4–6 µm tissue slices using a paraffin slicing machine (Leica-2135, Leica, Germany). After dewaxing, hydration, and heat-induced epitope retrieval using a Tris/ethylenediaminetetraacetic acid buffer (pH 9.0) in a pressure cooker, retinal tissue sections were inactivated with 3% hydrogen peroxide and then incubated with a primary antibody (rabbit cleaved caspase-3 [AC3]; 1:300, #9661s, Cell Signaling Technology, Danvers, MA, USA) overnight at 4°C, followed by a secondary antibody (PV-9001, ZSGB-BIO, Beijing, China) at 37°C for 1 h. AC3 immunoreactivity was detected via a chromogenic reaction using 3,3′-diaminobenzidine (ZLI-9017, ZSGB-BIO, Beijing, China) oxidization. Finally, apoptotic neurons (brown stained) were counted under a light microscope.

### 2.6 Terminal deoxynucleotidyl transferase dUTP nick-end labeling (TUNEL) assay

According to the protocol of the TUNEL AP kit (Roche Applied Science, USA), retinal tissue sections were permeabilized with proteinase K for 15 min after deparaffinization and hydration, followed by treatment with 3% hydrogen peroxide to inactivate endogenous peroxidase. The retinal tissue sections were incubated with a terminal deoxynucleotidyl transferase reaction mix for 60 min at 37°C and 4′,6-diamidino-2-phenylindole for 5 min at room temperature. A confocal fluorescence microscope (Leica TCS SP8; Leica, Germany) was used to capture TUNEL-positive cells (red stained).

### 2.7 Statistical analyses

AC3-positive cells were quantified from five discontinuous images that were randomly obtained using a light microscope (IX73, OLYMPUS, Japan), and TUNEL-positive cells were counted using a confocal fluorescence microscope (A1, Nikon, Japan) in each retina. The ratios of apoptotic cells in the GCL identified via AC3 immunoreactivity and the TUNEL assay were analyzed using Image-Pro Plus 6.0 (Media Cybernetics Company, USA). All data are presented as mean ± standard error of the mean. Data were analyzed using GraphPad Prism 5 or Origin 8.0. Differences between means of different groups were evaluated for statistical significance using the two-sample or unpaired Student’s *t*-tests to compare the frequency or amplitude of retinal waves following drug treatment. Non-normally distributed data were analyzed using the Mann–Whitney or Kruskal–Wallis tests. The level of statistical significance was set at *p* < 0.05.


**Informed consent:** Informed consent was obtained from all participants.
**Ethical approval:** This study was approved by the Ethics Committee of the Shanghai Sixth People’s Hospital, Shanghai Jiao Tong University (Approval No.: 2022-2-21), and complied with the guidelines for the Care and Use of Laboratory Animals published by the US National Institutes of Health and the Association for Research in Vision and Ophthalmology Statement for the Use of Animals in Ophthalmic and Vision Research.

## 3 Results

### 3.1 Muscimol blocked the synchronized spontaneous neural network electrical activity in the developing rat retinal GCL

**Figure 1:**
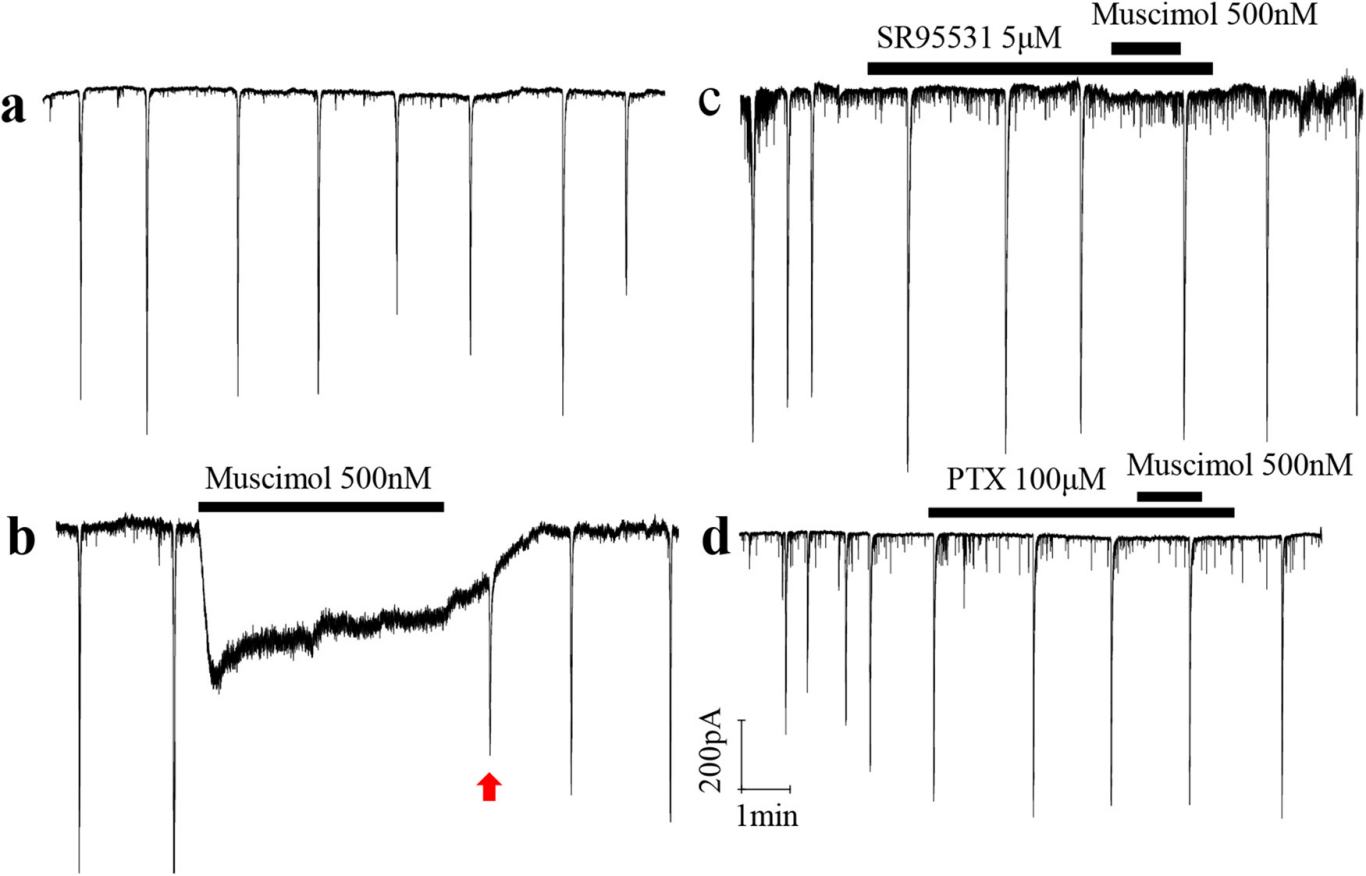
The effects of GABAa receptor agonist muscimol on the synchronized spontaneous neural network electricity activity in the developing rat retina GCL. The synchronized spontaneous neural network electricity activity were recorded by Whole-cell patch-clamp in P7 rat pups retina GCL (*n* = 6 retinas per group). (a) Synchronized spontaneous neural network electricity activity in the developing rat retinal GCL. (b) Puffing 500 nM muscimol around the recorded cell blocked the spontaneous and rhythmic neural network electricity activity transferring to the recording cell. The excitatory of the recoding cell restored and the neural network electricity activity were captured again (Red arrow mark) after stopping puffing muscimol. (c) and (d) The recording cell cannot be activated by muscimol puffing accompanied by bathing SR95531 (5 μM) or picrotoxin (PTX, 100 μM), antagonist of GABAa and GABAa/c respectively.

**Figure 2:**
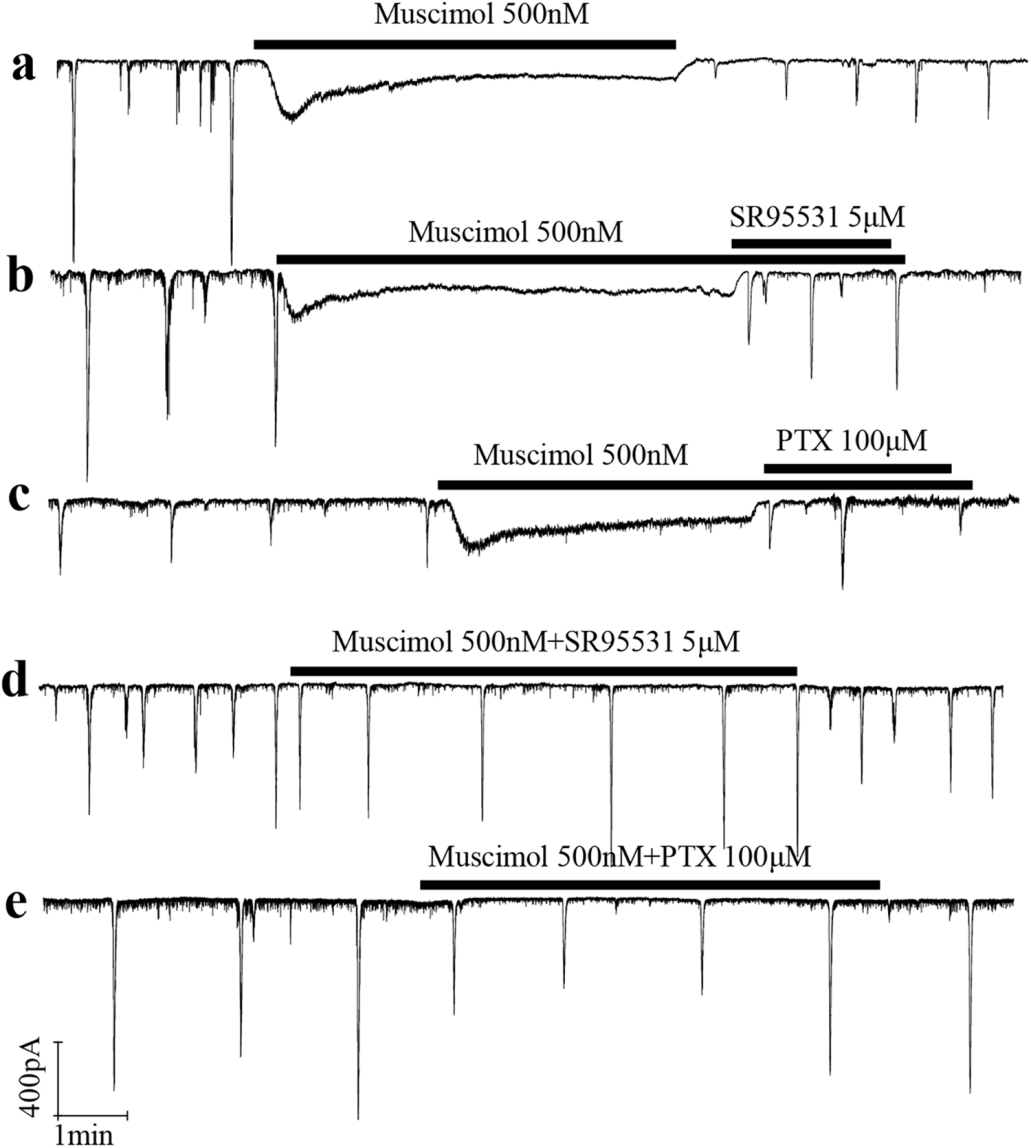
SR95531 and PTX reversed the effects of muscimol on the synchronized spontaneous neural network electricity activity. 5 μM SR95531 and 100 μM PTX (antagonist of GABAa and GABAa/c) reversed the effects of muscimol on the synchronized spontaneous neural network electricity activity in the developing rat retinal GCL (*n* = 6 retinas per group). (a) Bathing 500 nM muscimol blocked the spontaneous and rhythmic neural network electricity activity. (b) and (c) Both 5 μM SR95531 and 100 μM PTX reversed the effects of muscimol on the synchronized spontaneous neural network electricity activity. (d) and (e) The synchronized spontaneous neural network electricity activity cannot be blocked by bathing muscimol together with 5 μM SR95531 or 100 μM PTX.

Muscimol blocked the synchronized spontaneous neural network electrical activity in the developing rat retinal GCL. Using the whole-cell patch-clamp technique, we recorded the synchronized spontaneous neural network electrical activity in P7 rat retinal ganglion cells (Figure 1a). Application of muscimol (500 nM) completely blocked this activity (Figure 1b). This effect aligns with the reported transition of GABAa receptors from excitatory to inhibitory in the early developmental stage (<P7) [26,32]. GABAa receptors at this stage are excitable, allowing muscimol to depolarize retinal ganglion cells. However, sustained inward current by muscimol induced shunt conductance, causing a reversal in the resting potential and blocking the transfer of synchronized activity to the recorded cell (Figure 1b). After discontinuing muscimol application, the recorded cell gradually restored its ability to capture synchronized activity (red marks, Figure 1b). Additionally, bathing the GABAa antagonist SR95531 (5 μM) or the GABAa/c antagonist PTX (100 μM) prevented muscimol from activating the recorded cell (Figure 1c and d). In control experiments, muscimol completely blocked synchronized activity, but this effect was reversed by SR95531 or PTX in developing ganglion cells (Figure 2).

### 3.2 Muscimol induced neuronal apoptosis of the developing retinal GCL

**Figure 3:**
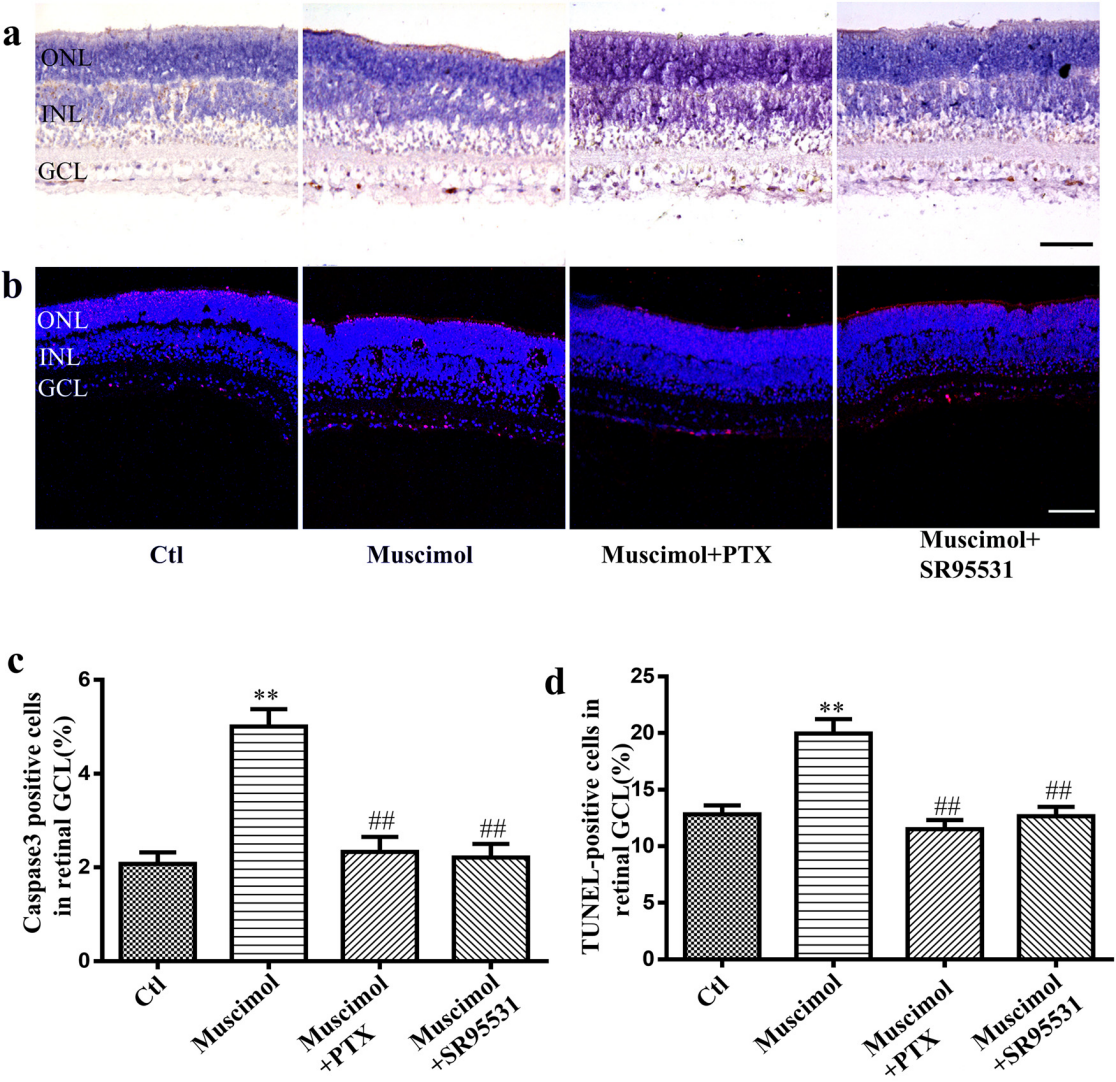
GABAa receptor agonist muscimol induced neuronal apoptosis in the developing retina GCL. Rat retinas were incubated with or without 500 nM muscimol for 5 hrs in the absence or presence of 5 μM SR95531 or 100 μM PTX (*n* = 5 retinas per group). (a) Photomicrograph of Caspase-3 positive cells (brown color) in the rat retinal GCL, scale bar, 50 μm. (b) Photomicrograph of TUNEL-positive cells (red color) in the rat retinal GCL, scale bar, 25 μm. (c) The ratio of Caspase-3 positive cells in the retinal GCL in different groups. (d) The percentages of TUNEL-positive cells in the rat retina GCL. Ctl: Control; Muscimol: 500 nM muscimol; Muscimol + PTX: 500 nM muscimol + 100 μM PTX; Muscimol + SR95531: 500 nM muscimol + 5 μM SR95531. Compared with the muscimol, the mean ratio of Caspase-3 positive cells are significantly different from the control and the Muscimol + PTX and Muscimol + SR95531 (** *p* < 0.01 compared with the control, ^##^ *p* < 0.01 compared with the Muscimol).

Treatment with muscimol led to a significant increase in apoptotic cells in the GCL of the developing rat retina, as determined by immunocytochemistry and the TUNEL assay. Furthermore, both SR95531 and PTX attenuated muscimol-induced neuronal apoptosis in the developing retinal GCL (Figure 3). These findings, in conjunction with those from Figure 2, confirm that activation of GABAa receptors can induce neuronal apoptosis in the developing nervous system by blocking synchronized spontaneous neural network electrical activity.

### 3.3 Effects of ethanol and dopamine on the synchronized spontaneous neural network electrical activity in the developing rat retina

**Figure 4:**
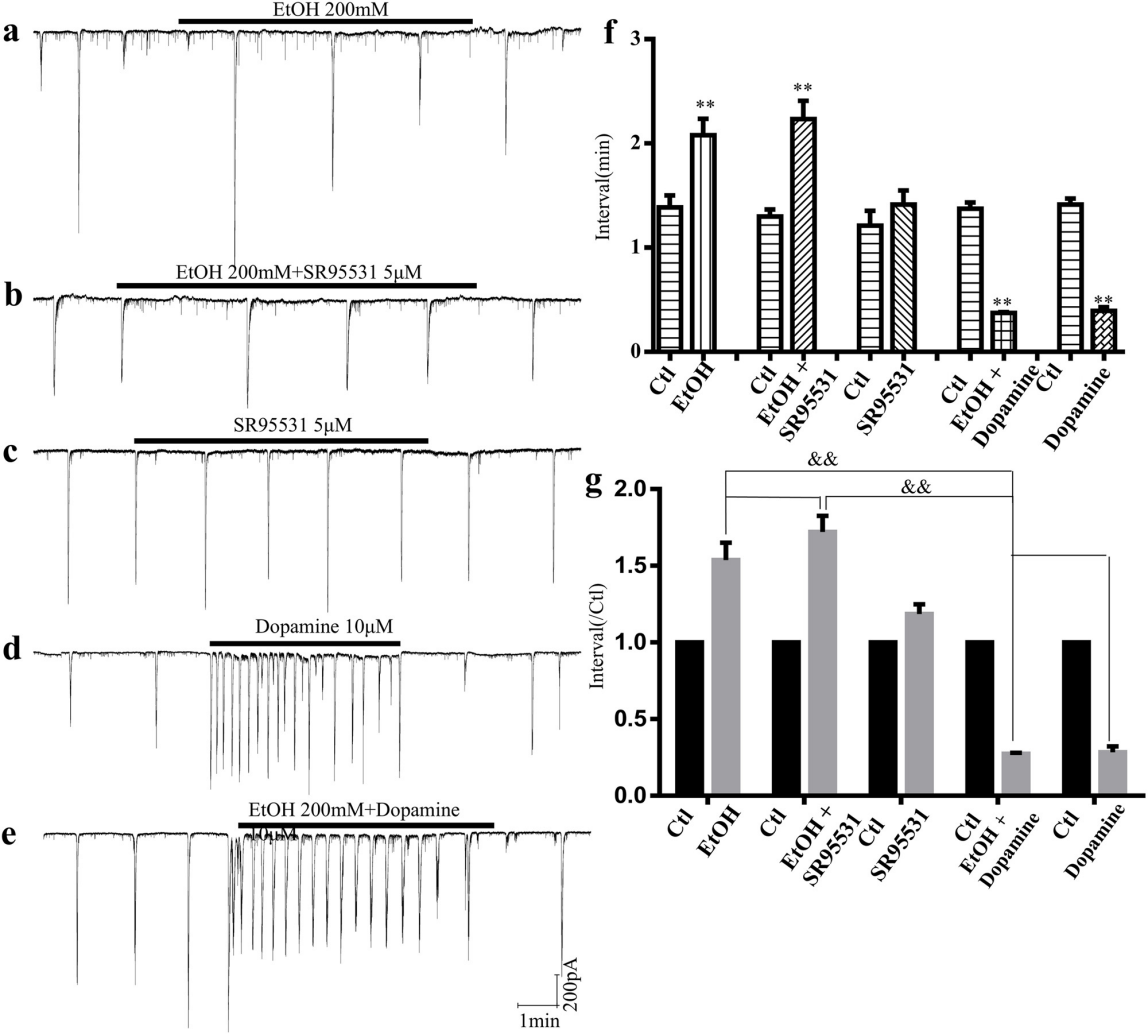
Effects of ethanol and dopamine on the synchronized spontaneous neural network electricity activity in the developing rat retina. Rat retinas were bathed with or without 200 nM ethanol in the absence or presence of 5 μM SR95531 or 10 μM dopamine (*n* = 6 retinas per group except the EtOH). (a) 200 nM ethanol decreased the frequency of the synchronized spontaneous network activity (*n* = 6 retinas). (b) 5 μM SR95531 cannot reverse the effects of ethanol on the synchronized spontaneous neural network electricity activity. (c) 5 μM SR95531 had no effect on the synchronized spontaneous neural network electricity activity. (d) and (e) 10 μM dopamine increased the frequency of the synchronized spontaneous network activity bathing with or without ethanol. (f) and (g) Interval of the synchronized spontaneous neural network electricity activity after bathing with or without ethanol in the absence or presence of SR95531 or Dopamine. Ctl: Control; EtOH: 200 nM ethanol; EtOH + SR95531: 200 nM ethanol + 5 μM SR95531; SR95531: 5 μM SR95531. EtOH + Dopamine: 200 nM ethanol + 10 μM dopamine; Dopamine: 10 μM dopamine. (** *p* < 0.01 compared with the control, ^&&^ *p* < 0.01 compared with the EtOH + Dopamine).

Our previous study demonstrated that ethanol induces neuronal apoptosis in the developing rat retina in a dose-dependent manner [33]. Here, we investigated the effects of ethanol on the spatiotemporal properties of synchronized spontaneous neural network electrical activity in the GCL of the developing rat retina. Ethanol (200 mM) significantly prolonged the interval of synchronized spontaneous network activity, increasing it from 1.39 ± 0.12 to 2.08 ± 0.16 min (control vs ethanol, *F* = 10, *p* < 0.01; Figure 4a). Importantly, this effect was not accompanied by changes in amplitude (data not shown). The GABAA antagonist SR95531 did not reverse the effects of ethanol on the synchronized spontaneous network activity. Under these conditions, the interval remained elevated at 2.23 ± 0.17 min compared to 1.30 ± 0.07 min in the control (control vs ethanol + SR95531, *F* = 6, *p* < 0.01; Figure 4b). Previous research has shown that dopamine increases the frequency of synchronized spontaneous network activity [34]. We assessed the impact of dopamine on ethanol-induced changes in the synchronized spontaneous network activity in the GCL. Dopamine significantly reduced the interval of spontaneous and rhythmic electrical activity in both the absence and presence of ethanol. Under control conditions, dopamine decreased the interval from 1.37 ± 0.05 to 0.35 ± 0.01 min (control vs dopamine, *n* = 6, *p* < 0.01; Figure 4d). Similarly, in the presence of ethanol, dopamine further reduced the interval from 1.37 ± 0.06 to 0.37 ± 0.01 min (control vs ethanol + dopamine, *n* = 6, *p* < 0.01; Figure 4e and f).

### 3.4 Effects of dopamine on ethanol-induced neuronal apoptosis in the developing rat retina

**Figure 5:**
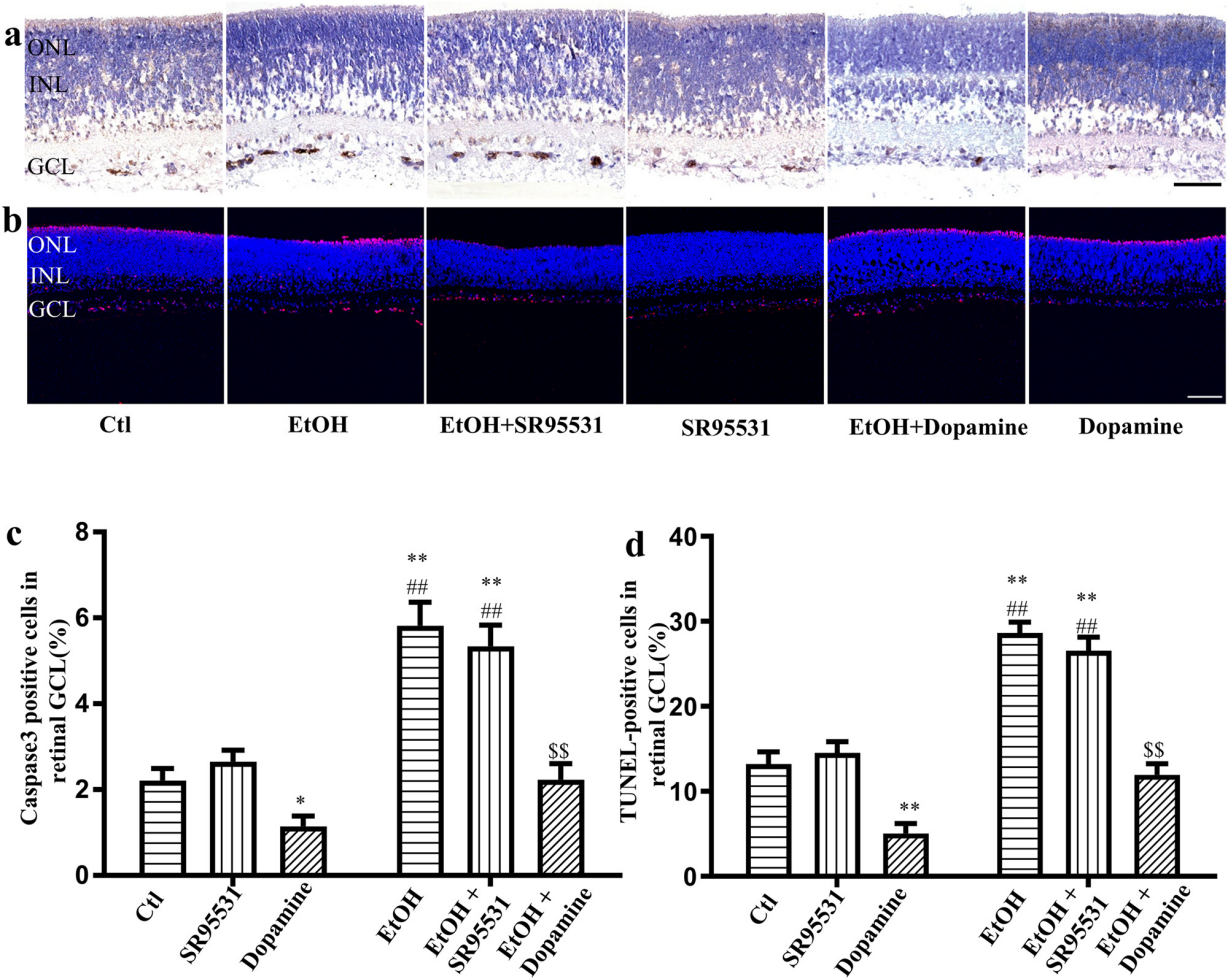
Effects of dopamine on the ethanol-induced neuronal-apoptosis in the developing rat retina. Rat retinas were incubated with or without 200 nM ethanol in the absence or presence of 5 μM SR95531 or 10 μM dopamine for 5 hrs (*n* = 5 retinas per group). (a) Photomicrograph of Caspase-3 positive cells (brown color) in the rat retinal GCL, scale bar, 50 μm. (b) Photomicrograph of TUNEL-positive cells (red color) in the rat retinal GCL, scale bar, 25 μm. (c) The ratio of Caspase-3 positive cells in the retinal GCL in different groups. (d) The percentages of TUNEL-positive cells in the rat retinal GCL. (* *p* < 0.05, ** *p* < 0.01 compared with the control, ^##^ *p* < 0.01 compared with the SR95531, ^$$^ *p* < 0.01 compared with the EtOH).

Ethanol-induced neuronal apoptosis in the GCL of developing rat retina was similar to that observed in our previous study [33]. In this study, after 5 h of exposure to 200 mM ethanol, neuronal apoptosis occurred significantly in the developing rat retinal GCL. The ratio of neuronal apoptosis was upregulated from 2.21 ± 0.29 to 5.82 ± 0.55% (Figure 5a and c, *n* = 25, *p* < 0.01) after 5 h of incubation with ethanol by immunochemistry and 13.23 ± 1.42 to 28.65 ± 1.25% (Figure 5b and d, *n* = 25, *p* < 0.01) by the TUNEL assay. SR95531 did not alleviate ethanol-induced or physiological neuronal apoptosis, by both immunochemistry and the TUNEL assay. Dopamine, which can increase the frequency of the spontaneous and rhythmic network electrical activity, attenuated the physiological neuronal apoptosis from 2.21 ± 0.29 to 1.15 ± 0.024% (*n* = 25, *P* = 0.03) by immunochemistry and 13.23 ± 1.42 to 5.06 ± 0.49% (*n* = 25, *p* < 0.01) by the TUNEL assay (Figure 5). In addition, the ratio of ethanol-induced neuronal apoptosis was downregulated from 5.82 ± 0.55 to 2.23 ± 0.37% (*n* = 25, *p* < 0.01) and 28.65 ± 1.25 to 11.95 ± 1.32% by immunochemistry and the TUNEL assay (*n* = 25, *p* < 0.01), respectively, after co-incubation with dopamine (Figure 5).

## 4 Discussion

In this study, we observed that muscimol inhibited synchronized spontaneous neural network electrical activity and stimulated neuronal apoptosis in the developing rat retinal GCL. This finding confirms that blocking synchronized spontaneous neural network electrical activity can induce neuronal apoptosis in the developing nervous system. Ethanol disrupts the synchronized spontaneous neural network electrical activity by altering its spatiotemporal properties, which may be associated with ethanol-induced neuronal apoptosis in the developing rat retinal GCL. Additionally, SR95531 was unable to reverse the effect of ethanol on synchronized spontaneous neural network electrical activity and neuronal apoptosis. Conversely, dopamine increased the frequency of synchronized spontaneous neural network activity, both in the presence and absence of ethanol, and attenuated both physiological and ethanol-induced apoptosis.

During the early stages of neural system development, the sequential activation of GABA, NMDA, and ACh receptors or their subtypes regulates spontaneous and rhythmic neural network electrical activity [18–20]. This study revealed that ethanol-induced neuronal apoptosis was partially mediated by disrupting the spatiotemporal properties of synchronized spontaneous neural network electrical activity. Although ethanol can block NMDA receptors and activate GABA receptors [35], previous studies have shown that the effects of ethanol on GABAa receptors are associated with an enhancement of GABA-stimulated Cl^–^ flux, increased GABA neuronal activity, and GABA release [36–38]. This may explain why ethanol cannot completely block neural network electrical activity, unlike muscimol, by reversing the resting potential. Furthermore, the synchronized spontaneous neural network electrical activity transitions from a cholinergic drive to a glutamatergic drive, primarily occurring between postnatal Days 7–9 (P7–P9) in rats (nicotinic ACh receptors for <P7 and NMDA glutamate receptors for >P9). Therefore, ethanol cannot fully block network electrical activities by solely blocking NMDA receptors. The precise mechanism by which ethanol disrupts the spatiotemporal properties of synchronized spontaneous neural network electrical activity remains to be explored in future studies.

A previous study demonstrated that the spatiotemporal properties of neural network activity are regulated by a cAMP/PKA cascade [34]. In this study, dopamine effectively attenuated ethanol-induced neuronal apoptosis by stimulating neural network electrical activity. Additionally, our prior research indicated that dopamine reduces ethanol-induced apoptosis through the cAMP/PKA pathway [33]. Consequently, it can be concluded that dopamine attenuates ethanol-induced neuronal apoptosis by stimulating neural network electrical activity via activation of the cAMP/PKA cascade. However, the exact molecular mechanisms underlying this process require further investigation.

## 5 Limitations and future directions

Several limitations exist in the present study. First, the exact mechanism of ethanol-induced disruption of the spatiotemporal properties of neural network electrical activity was not explored. Second, the study did not investigate how dopamine increases neural network electrical activity, thereby exerting a protective effect. Furthermore, this study focused exclusively on the neuroapoptosis induced by ethanol treatment at P7 in the rat GCL. Future research is needed to explore the effects of ethanol and dopamine on other regions of the developing brain.

## 6 Clinical implications

These findings have potential translational implications for clinical practice. Specifically, targeting dopamine pathways may offer a therapeutic strategy to mitigate ethanol-induced neuronal damage during early development. Given the protective effects of dopamine on neuronal apoptosis and neural network activity, pharmacological interventions or therapies that enhance dopamine signaling could be developed to treat or prevent ethanol-induced neurotoxicity in vulnerable populations, such as infants or individuals with fetal alcohol spectrum disorders. Future studies should explore the feasibility and efficacy of such interventions in preclinical models and clinical settings.

## 7 Conclusion

In this study, we found that ethanol induced neuronal apoptosis in the developing rat retinal GCL by discombobulating the spatiotemporal properties of the synchronized spontaneous neural network electrical activity. Dopamine increased the frequency of the neural network electrical activity and attenuated ethanol-induced apoptosis.
